# Odorant Binding Proteins: a key player in the sense of smell

**DOI:** 10.6026/97320630014036

**Published:** 2018-01-31

**Authors:** Govindaraju Archunan

**Affiliations:** 1 Centre for Pheromone Technology (CPT), Department of Animal Science, Bharathidasan University, Tiruchirappalli- 620 024. TN, India;

**Keywords:** olfaction, pheromone, smell, OBP, olfactory receptor

## Abstract

Olfaction is an important mechanism by which humans and animals communicate with environment. Odorant-binding proteins (OBPs) play crucial role in the olfactory mechanism. Here, we briefly discuss about the role of OBPs and their importance in industrial, pest management and therapeutic developments.

## Background

Our natural environment has several thousands of different odors. Animals and humans have sophisticated system to detect, discriminate and interpret those odors. This process is called as olfaction or "the sense of smell" mechanism [1]. Humans and animals use olfactory mechanism to communicate with environment, to locate food sources, to identify mates, to avoid predators, and to provide both sensual pleasure as well as warnings of danger. Apart from those important roles, olfaction also plays other roles in the lives of humans and animals. A recent study in mouse suggests that loss of ability to smell may signal early Alzheimer's disease [2]. Another study shows that olfaction is a key factor in long-distance oceanic navigation in birds [3]

Olfaction is one of the oldest sensory modalities known in evolution. However, the precise olfactory mechanism is not fully understood and is still the subject of research. The very first step in the olfaction is to deliver odorant molecules from the environment to the olfactory receptors. Humans and animals have special proteins called odorant-binding proteins (OBP). These proteins binds to the odorant molecule dispersed in the environment and transport them to olfactory receptors, which are located in olfactory sensory neurons in the olfactory epithelium in the nose of humans, and in the vomeronasal organ in animals [4]. Vertebrate OBPs are members of large lipocalin family and shares eight stranded beta barrel. Insects have two types OBPs: general odorant-binding proteins (GOBPs) and the pheromonebinding proteins (PBPs). They are completely different from their vertebrate counterpart both in sequence and three-dimensional folding. Insect OBPs contains alpha helical barrel and six highly conserved cysteines. Another class of putative OBPs, named chemosensory proteins (CSPs) has been reported in different orders of insects, including Lepidoptera. In spite of the sequence and structural difference, their general chemical properties indicate similar functions in olfactory transduction. They also function to remove used odorants for breakdown and free up the receptor to detect other molecules.

OBP has received much attention due to its biotechnological and therapeutic importance. Mosquitoes transmitting diseases such as malaria, dengue etc, create serious threats to human population all over the world. Each year, nearly 700 million people are affected by disease transmitting mosquitoes [5]. A better understanding of the structural biology of mosquito odorant binding proteins may pave the way for the development of environmentally-friendly mosquito attractants and repellents, which may ultimately contribute to reduction of mosquito biting and disease transmission.

The application of an odorant binding protein for odor control and fragrance delayed release from a textile surface was explored in the study performed by Silva et al. 2014 [6]. The structural architectures of OBPs are extremely stable to temperature, organic solvents and proteolytic digestion. These characteristics make OBPs suitable elements for fabricating biosensors to be used in the environment, as well as for other biotechnological applications [7, 8]. Detailed study of olfaction will aid in finding treatments for those who have lost their sense of smell as a result of age, disease, or sensory damage.

Several experimental and bioinformatics studies to identify and analyse OBPs, have been reported. Advances in genome sequencing technologies allow researchers to study OBP genes and their expression patterns at the genome level have been reported [9, 10]. A complete understanding of structure and function of OBPs and olfactory mechanism will help us to develop several important therapeutic and industrial applications in future.

## Conclusion

Olfaction is essential mechanism in human and animals to locate food, identify mates, individual identification, and avoid toxic substances and so on. Odorant binding proteins play crucial role in olfactory mechanism. Detailed study of OBPs and their role in olfactory mechanism is important to develop industrial, agricultural and therapeutic applications. Currently, several promising approaches on genomics and proteomics are on going to gain more information about the function of OBPs.
